# The Economic Impact of Paediatric Integrated Care in Primary Care: A Rapid Review

**DOI:** 10.5334/ijic.10232

**Published:** 2026-11-06

**Authors:** Hamish McCartney, Sandra Davidson, Ann-Maree Duncan, Harriet Hiscock, Raghu Lingam, Lena Sanci

**Affiliations:** 1 Melbourne Medical School, Faculty of Medicine, Dentistry and Health Sciences, University of Melbourne, Victoria, Australia; 2 Department of General Practice, Faculty of Medicine, Dentistry and Health Sciences, The University of Melbourne, Melbourne, Victoria, Australia; 3 Health Services and Economics, Murdoch Children’s Research Institute, Victoria, Australia; 4 Department of Paediatrics, The University of Melbourne, Melbourne, Victoria, Australia; 5 School of Clinical Medicine, University of New South Wales, Sydney, New South Wales, Australia

**Keywords:** integrated care, paediatric, primary care, general practice, cost effectiveness, cost analysis

## Abstract

**Background:**

Health systems face mounting pressure to deliver equitable, cost-effective care amid rising disease burden and healthcare costs. Escalating paediatric out of pocket costs and other barriers to access, highlight the need for innovative care models. While integrated care shows promise for paediatric condition management, economic evidence remains limited.

**Methods:**

Searches were conducted between 2010 to 2025 on MEDLINE, EMBASE and CINAHL using search terms “integrated care”, “paediatrics”, “primary care” and “cost-effectiveness”. Studies were included if they reported a cost-related outcome of a paediatric integrated care program delivered in primary care. Studies were assessed for methodological reporting quality using the CHEERS guideline.

**Results:**

Nine studies met inclusion criteria. Interventions comprised care coordination models (n = 4), MDT-based care (n = 3) and co-consultation models (n = 2). Seven studies reported a favourable economic outcome with six being cost-effective, and one showing cost-savings. Care coordination models showed the strongest evidence for economic impact and clinical effectiveness.

**Conclusion:**

Integrated paediatric primary care models, particularly care coordination models, are economically favourable. Complex systems-level interventions show limited financial benefits; however, they are difficult to evaluate without understanding the local context of the intervention. Future research should prioritise improving the quality of economic evaluations and inclusion of societal perspectives into economic evaluation.

## Introduction

A major challenge for healthcare systems around the world is the rising cost of providing accessible, quality healthcare in the face of an increasing burden of disease [1,2]. A key component of a sustainable health care system is a strong primary health care system where conditions are well managed, thus reducing the need for more expensive specialist and hospital-based care [3,4]. With constrained health budgets both in Australia and globally, there is an increased need to ensure that models of care are both equitable and cost-effective, whilst also being clinically effective [5].

In Australia, the healthcare system is increasingly struggling to adequately address the care needs of the paediatric population [6,7]. There has been an increasing burden of disease for paediatric conditions which has led to rising demand on secondary and tertiary sectors [8,9,10]. Children aged 0–4 are over-represented in emergency departments (EDs), having the second highest rates of presentation of all age groups [11]. Evidence shows that many of these presentations are low urgency and could be appropriately managed in primary care [12,13]. There are also increasingly long wait times for specialist outpatient care [6,7,14]. The cost of paediatric hospitalisations to the healthcare system is significant and has not shown any reduction over time [4]. The increasing burden of chronic paediatric conditions, avoidable ED presentations, and associated healthcare costs of managing care in the tertiary sector highlights the need to strengthen the capacity of the primary care sector.

Integrated care has been identified by the World Health Organization (WHO) as being able to potentially reduce health care costs whilst improving quality of care [5]. A pragmatic definition divides integrated care into vertical, horizontal and longitudinal frameworks [15]. Vertical integration is between primary and secondary/tertiary healthcare, horizontal integration is between health and the social contexts (such as education and social care), and finally, longitudinal care is ensuring age-appropriate care across the lifespan [15]. They highlight collaboration between medical, behavioural and allied health professionals to ensure seamless communication and care provision across providers and care settings – a response to a fragmented healthcare system [16].

Integrated care has been identified, particularly in paediatric populations, to improve health outcomes by enabling more effective management of conditions within the primary care sector [17,18,19]. However, the evidence for the economic sustainability of integrated care for paediatric populations is limited. A 2020 systematic review of 18 randomised controlled trials (RCTs) of integrated care interventions for populations under the age of 18 identified only five studies that examined cost savings and/or cost-effectiveness [18]. Of these, three interventions reported a positive economic result [18]. The review found limited but encouraging evidence supporting integrated care as offering improvements to child quality of life (QoL) [18]. A dedicated review of the latest economic evidence is needed to support decisions on the adoption and implementation of paediatric integrated care programs.

The aim of this rapid review is to understand the economic costs and benefits of paediatric integrated care programs delivered in primary care. The review builds on previous work [18] by including more recent studies and broadening the inclusion criteria from RCTs to include all study designs. The review also will provide an in-depth analysis of the economic methods and findings used in each study. The primary question of interest is: what is the economic impact of integrated care programs in primary care for paediatric patients (aged 0–18)? A secondary question is: how do the economic outcomes relate to the reported clinical or health service outcomes of the included interventions?

## Methods

The review was conducted as a rapid review. Adaptations included restricting database searches to three sources, excluding non-English language studies, one reviewer used for data extraction and quality appraisal and limiting grey literature searching to hand searching of reference lists. A rapid review methodology was selected to provide timely evidence synthesis for health policy and planning decisions, with an accepted trade-off in comprehensiveness for speed.

A search was conducted across three databases: Medline, EMBASE Ovid and CINAHL for English language articles published between January 1^st^, 2010, and March 5^th^, 2025. The search was limited to studies published from January 1, 2010, onwards to capture contemporary integrated care models that reflect current healthcare delivery practices and policy frameworks. The search strategy combined four concept areas using Boolean operators: “integrated care” AND “paediatrics” AND “primary care” AND “cost-effectiveness,” with appropriate synonyms for each concept. The full search strategy is available as a supplementary file. The search was conducted on March 6, 2025. A manual search was also conducted of the reference list of all screened full text articles to identify eligible articles that were missed in the database searching. Articles that were excluded in full text review also underwent a manual search to find related articles as there were several abstracts and protocols present in full text screening. Initial scoping searches of additional specialized databases (EconLit, NHS Economic Evaluation Database and the Paediatric Economic Database Evaluation) were conducted but yielded no additional eligible studies beyond those identified in the main database searches. Table 1 outlines the inclusion and exclusion criteria used. Integrated care was defined in the review as involving at least two different health professionals working collaboratively, with cross-sector involvement via vertical integration, horizontal integration, or both.

**Table 1:** Eligibility criteria.


*Inclusion criteria*	Integrated care program delivered in primary care and involved vertical and/or horizontal integration Study participants aged between 0–18 and ongoing physical or mental health condition Included > one cost-related outcome (i.e., full or partial economic evaluation) Conducted in high income countries and intervention could be generalizable to the Australian healthcare system All experimental study designs Original research

*Exclusion criteria*	Paediatric outcomes could not be disaggregated from adult outcomes. Full text not available Conference poster presentations, opinion, commentary, perspective, editorial, books/dissertations Non-English language


Search results were imported into Covidence with duplicates automatically and manually removed. Two reviewers (HM and SD) screened title and abstract for eligibility using pre-defined criteria. Papers were then independently read in full by two reviewers (HM and SD) with disagreements resolved through discussion. A single reviewer (HM) scanned reference lists of all articles that underwent full text review to identify additional relevant studies. Data from included articles was extracted by a single reviewer (HM) using a standardised template that had been pre-approved by the research team. Due to the diverse nature of the interventions, a narrative synthesis of studies was performed. Data that was analysed included: details of interventions, summary of the primary outcomes, summary of economic evaluation designs and summary of economic outcomes as described in the studies.

Methodological reporting quality was assessed by HM using the 28 item Consolidated Health Economic Evaluation Reporting Standards (CHEERS) 2022 guideline [20]. Each publication was assessed by scoring individual items as ‘1’ if the criterion was fulfilled, ‘0’ if it was not fulfilled, or ‘NA’ if the item was not applicable. The overall quality score for each study was calculated as the percentage of applicable items that were fully met.

People with lived experience of integrated care were not involved in the completion of the review due to time and resource restraints.

## Results

### Characteristics of studies

The database search identified 246 unique records (Figure 1). Of these, 54 were selected for full-text screening and six were included in the review. A hand search of reference lists of included papers identified three more eligible studies, bringing the total studies included to nine.

**Figure 1:**
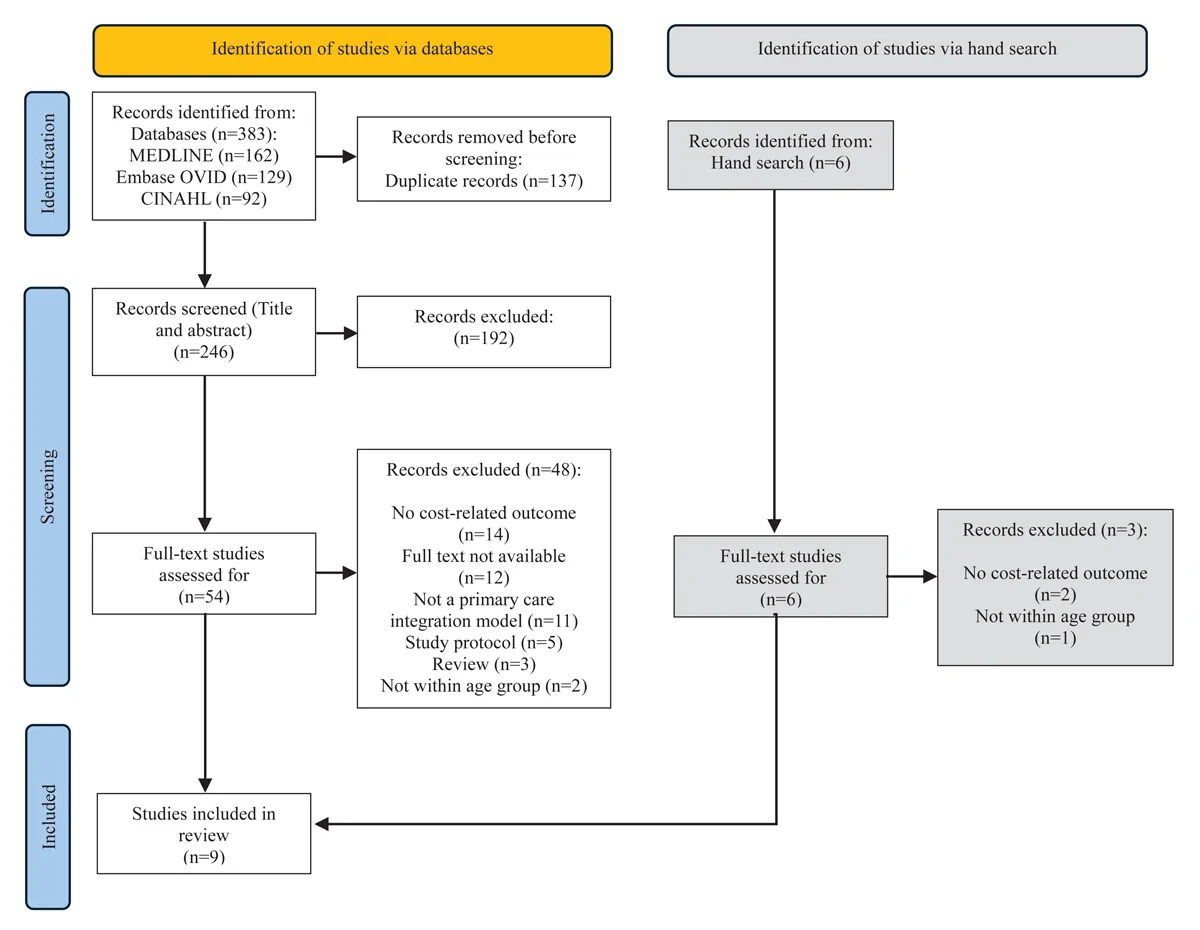
PRISMA diagram.

A summary of studies and their primary outcome is presented in Table 2. Four of the nine studies were conducted in the UK [21,22,23,24], three in the USA [25,26,27], and two in Australia [28,29]. Five of the studies were randomised controlled trials (RCTs) (one was a pilot trial) [22,24,26,27,28], three were pre-post pilot studies [21,25,29] and one was a decision-analytic model from a pilot study [23]. Three of the nine studies had a sample size greater than 500. The earliest study was published in 2012 and only one study (DOCC) was also found in Wolfe’s systematic review and meta-analysis [18].

**Table 2:** Summary of studies evaluating integrated care with their primary outcome.


AUTHOR/YEAR/COUNTRY	INTERVENTION NAME	CATEGORY OF INTEGRATION	PRIMARY OUTCOME	DESCRIPTION OF INTERVENTION	CONDITION TREATED	RESULT OF PRIMARY OUTCOME	STUDY DESIGN/COMPARISON GROUP	POPULATION SETTING/SAMPLE SIZE

**Carter** **2022** [28] **Australia**	Integrated children’s clinic care (ICCC)	Care coordination	Child quality of life [30]	Children were allocated an allied health liaison officer (AHLO) for care coordination with tasks including attending primary care appointments, health literacy education and support navigating primary and secondary health systems	ADHD, anxiety, developmental delay, behavioural conditions	No difference in child quality of life between groups (p > 0.05) [30]	RCT TAU	0–16 years 81 participants

**Cullen** **2017** [21] **UK**	Learning Together (LT)	Co-consultation	Guideline adherence for common conditions (constipation, asthma, feverish illness and eczema) [31]	Co-consultations between a GP registrar and a paediatric registrar in a general practice setting	All paediatric presentations	Significant improvement in guideline adherence during and after the intervention (p < 0.05) [31]	Pre-post study (Pilot) N/A	Integrated GP-paediatrician clinic 848 children consulted in 145 LT clinics

**Hiscock** **2020** [29] **Australia**	Strengthening Care for Children Pilot (SC4CP)	Co-consultation	ED presentations, referral to hospital clinics.	Co-consultation between a GP and paediatrician in a general practice setting. The model also included monthly case study discussions and telephone/email education support for GPs from paediatricians	All paediatric presentations	Reduction in ED presentations and referral to hospital clinics (p > 0.05)	Pre-post study (Pilot) N/A	0–17 years Five Integrated GP-paediatrician clinics 624 co-consults

**Hollinghurst** **2014** [22] **UK**	Primary Care-Care of Childhood Obesity (PC-COCO)	Paediatric MDT in primary care	Change in BMI standard deviation score	A multidisciplinary hospital obesity clinic was replicated in a primary care setting run by primary care nurses alongside dieticians and exercise specialists	Obesity	Significant reduction in BMI SDS in primary health group (p < 0.05)	Pilot randomized trial TAU	5–16 years 143 children

**Melyda** **2024** [23] **UK**	Sparkbrook’s Children Zone (SCZ)	Paediatric MDT in primary care	ED presentations	An integrated clinic with GPs working with paediatricians, paediatric nurses, family support workers, mental health workers to improve management of common conditions and social care intervention	All paediatric presentations	Reduction in ED presentations (p > 0.05)	Decision-analytic model (Pilot) N/A	0–16 years N/A [32]

**Soley-Bori** **2021** [24] **UK**	Children and young people’s health partnership model (CYPHP)	Paediatric MDT in primary care & Co-consultation	ED presentations & child quality of life scores [33]	Two types of services – targeted care for children and young people (CYP) with tracer conditions (asthma, eczema, epilepsy, constipation); universal care for CYP with any condition. Tracer conditions: tailored MDT including specialist children’s nurses, pharmacist, mental health workers, school nurses, paediatrician, GP and child and adolescent psychiatrist Universal care: Co-consultation between a GP and a paediatrician (virtually linked) in a general practice setting [34].	All paediatric presentations	No difference in ED presentations or child quality of life (p > 0.05) [33]	Cluster RCT EUC	0–16 years. 70 GP clinics formed 23 virtual clusters. 12 clusters assigned to CYPHP 1,731 children

**Woods** **2012** [25] **USA**	Community Asthma Initiative (CAI)	Care coordination	Reduction in hospitalisations & ED presentations based upon parent-completed interviews	Nurse care coordination between primary care and referral services, and home visits for asthma education, environmental assessment	Moderate to severe asthma	Significant reduction in hospitalisations and ED presentations (p < 0.05)	Pre-post study (Pilot) N/A	2–18 years 283 participants

**Wright** **2016** [26] **USA**	Reaching out to Adolescents in Distress (ROAD)	Care coordination	Depression symptoms [35]	Individuals with depression were recruited from primary care and allocated a depression care manager (DCM). DCMs could: deliver psychotherapy, follow-up with patients and families to assess treatment adherence and communicate information back with primary care clinician. Weekly team meetings also took place with a psychiatrist, psychologist and paediatrician [35].	Depression	Significant reduction in depression symptoms (p < 0.05) [35]	RCT TAU	13–17 years 101 participants

**Yu** **2017** [27] **USA**	Doctor’s Office Collaborative care (DOCC)	Care coordination	Child mental health symptoms & parental stress [36]	Mental health clinicians were trained as case managers to screen and treat behavioural conditions and work with primary care paediatricians to manage care.	Paediatric behavioural problems and ADHD	Significant improvement in both child mental health symptoms and parental stress (p < 0.05) [36]	RCT EUC	5–12 years 321 participants


MDT: multi-disciplinary team; TAU: treatment as usual; EUC: enhanced usual care.

The conditions targeted in each study varied with four focusing on all general paediatric presentations [21,23,24,29], three directed at mental health/behavioural conditions (i.e. depression, attention-deficit/hyperactivity disorder (ADHD), developmental delay) [26,27,28], one on asthma [25] and one on obesity [22]. There were three broad categories of integrated care interventions: care coordination (n = 4) [25,26,27,28], paediatric multidisciplinary team-based primary care (MDT) (n = 3) [22,23,24], and co-consultation between a general practitioner and a paediatrician (n = 2) [21,29].

#### Care coordination (n = 4)

Care coordination programs involved a trained worker being allocated to patients to aid navigation of primary and secondary health systems. The Australian study, Integrated children’s clinic care (ICCC), provided an allied health liaison officer (AHLO) to patients with mental health and/or neurodevelopmental conditions [28]. The role of the AHLO was to assist patients and families to navigate the primary and secondary health systems (e.g. attending appointments, supporting health literacy) [28]. One USA study, Community Asthma Initiative (CAI), had a nurse care coordinator who provided home visits and aided in referral between services for children with moderate to severe asthma [25]. The Reaching out to Adolescents in Distress (ROAD) study in the USA had a depression care manager who could deliver therapy, follow-up with patients and communicate with primary care clinicians [26]. The Doctor’s Office Collaborative Care (DOCC) study, also set in the USA, had a mental health clinician, trained as a case manager, who could treat and work alongside primary care paediatricians for children with paediatric behavioural problems and/or ADHD [27].

#### Multidisciplinary Primary Care Teams (n = 3)

The MDT interventions took different forms. The South London based, Children and Young People’s Health Partnership model (CYPHP) involved two pathways: one for managing long term tracer conditions (asthma, eczema, epilepsy and constipation), and one for children with general paediatric problems [24,33]. The first pathway involved a tailored MDT of allied heath staff (e.g. paediatric nurses, pharmacists, mental health workers, and school nurses) working alongside a GP [24,33]. The second pathway included co-consultation between GPs and paediatricians. Although CYPHP included both a paediatric MDT and co-consultation in its intervention design, we classified it under the paediatric MDT model, as the economic evaluation was based on the MDT component [24]. Sparkbrook’s Children Zone (SCZ), based in Birmingham UK, also involved an MDT of allied health staff (paediatric nurses, family support workers and mental health workers) working alongside a GP for all paediatric presentations [23]. The Primary Care-Care of Childhood Obesity model (PC-COCO), also in the UK, replicated a hospital obesity clinic in a primary care setting run by nurses alongside dieticians and exercise specialists, for children with obesity (body mass index (BMI) greater than the 98^th^ centile) [22].

#### Co-consultation (n = 2)

The Learning Together (LT) intervention in the UK involved a GP registrar co-consulting with a paediatric registrar for all paediatric presentations [21]. The Strengthening Care for Children pilot (SC4CP), in Australia involved a GP co-consulting with a paediatrician alongside monthly case study discussions and educational support for GPs [29].

### Economic analyses

Table 3 summarises the economic evaluations reported in each study. Three studies conducted a cost-utility analysis, three conducted a cost-effectiveness analysis (two based on primary data, the third from modelling from a pilot study) and three studies undertook a cost-analysis with each study reporting results in a different way. All nine studies adopted a health system perspective, with two of these also including a societal perspective in the economic evaluation [24,28]. Six studies applied a time horizon of 12 months [21,22,24,26,28,29], two studies 24 months [25,27] and one study 30 days [23].

**Table 3:** Summary of economic evaluations.


AUTHOR/YEAR/INTERVENTION	PERSPECTIVE	DATA SOURCE	TYPES OF COSTS MODELLED	TYPE OF ANALYSIS	RESULTS REPORTED AS	BASE YEAR. CURRENCY	TIME HORIZON	RESULT

**Full Economical Evaluations (n = 6)**								

**Carter** **2022** [28] **ICCC**	Health system	RCT data from families and caregivers	Intervention delivery (staff time), hospital admissions, ED visits, GP visits,	Cost-utility analysis	ICER Net cost impact	2021. AUD	12 months	ICER of $548 AUD per QALY gained.

	Societal	RCT data from families and caregivers	Included caregiver productivity losses	Cost-utility analysis	ICER Net cost impact	2021. AUD	12 months	ICER dominant – more effective and less costly than standard care

**Hollinghurst** **2014** [22] **PC-COCO**	Health system	Primary data – RCT; clinic service records	Intervention delivery (staff time), follow-up visits, healthcare resource use;	Cost-effectiveness analysis	ICER	2011. GBP	12 months	ICER of £175 GBP per 0.1 BMI standard deviation score lost.

**Melyda** **2024** [23] **SCZ**	Health system	Modelled economic evaluation based on 30-day pilot service logs and local authority data	MDT triage and consultation time, GP visits, hospital attendances (modelled); costs drawn from NHS unit cost data.	Cost-effectiveness analysis (modelled)	ICER	2022–23. GBP	30 days	The modelled ICER dominated – reduced hospital use and cost

**Soley-Bori** **2021** [24] **CYPHP**	Health system	Primary data – RCT, health and social care use surveys	Intervention costs, health care utilisation (GP, hospital, community services), out-of-pocket expenses	Cost-utility analysis	ICER	2019–20. GBP	12 months	ICER of £45,586 GBP per QALY at 12 months

	Societal	Primary data – RCT, health and social care use surveys	Caregiver time and work absences	Cost-utility analysis	ICER	2019–20. GBP	12 months	ICER of £22,966 GBP per QALY at 12 months

**Woods** **2012** [25] **CAI**	Health system	Primary data – program data, hospital billing and claims records	Program delivery (nurse visits, education, case management), emergency department visits, hospital admissions.	Cost-effectiveness analysis	Cost savings per patient	2006. USD	24 months	Cost-savings, with $1,298 USD saved per child over two years

**Wright** **2016** [26] **ROAD**	Health system	Primary data – RCT, family and service use questionnaires	Intervention delivery (care manager, clinical time), health service use (mental health, hospital, primary care), prescription costs	Cost-utility analysis	ICER	2014. USD	12 months	ICER $18,239 USD per QALY gained

**Partial Economic Evaluations (n = 3)**								

**Cullen** **2017** [21] **LT**	Health system	Cost-modelling study – based on training implementation assumptions	GP training costs, cost of GP time, modelled NHS savings per child managed in primary care (vs. secondary care); no direct health service use data	Cost analysis	Cost difference per clinic	Not reported. GBP	12 months	Intervention cost an additional £37 GBP per clinic compared with usual training.

**Hiscock** **2020** [29] **SC4CP**	Health system	Primary data with modelled cost-savings projections	GP and paediatrician time, clinic overheads, community health resources; modelled potential savings in referral reductions and resource use	Cost analysis	Net cost per child	Not reported. AUD	12 months	Model cost estimates were $172 AUD above usual care per child seen in the co-consultations.

**Yu** **2017** [27] **DOCC**	Health system	Primary data – RCT, intervention logs, insurance claims for follow-up	Mental health clinician time, training/supervision costs, community mental health claims, medication, and service use at 6–24 months	Cost analysis	Cost per patient treated Cost savings in community mental health care	2010. USD	24 months	Mixed results Cost savings at 6 and 12 months $497 USD per participant at 6 months post-intervention (p < 0.05) $773 12 months post-intervention (p < 0.05) Not cost savings at 18- and 24-months post-intervention (p > 0.05)


AUD: Australian Dollar; GBP: British Pound; USD: US Dollars; ICER: Incremental cost-effectiveness ratio; CEA: cost-effectiveness analysis; CUA: cost-utility analysis; QALY: quality-adjusted life year.

### Economic evaluation results

#### Care Coordination

ICCC and ROAD completed cost-utility analyses (results reported as costs per QALY gained) [26,28], CAI completed a cost-effectiveness analysis (cost savings per patient) [25], and DOCC completed a cost analysis (cost savings per patient) [29]. The ICCC reported a 73% likelihood of being cost-effective from a health systems perspective and a 78% likelihood when applying a societal perspective (including caregiver productivity losses) based on 10,000 probabilistic simulations and a $50,000 AUD/QALY gained willingness to pay (WTP)) [28]. The ROAD model reported favourable cost-effectiveness with an incremental cost-effectiveness ratio (ICER) of $18,239 USD per QALY, which was below the WTP threshold of $50,000 USD/QALY gained [26]. The CAI study reported a cost savings (derived from reduced emergency department visits and hospitalisations) per patient with $1,298 USD saved per child over two years [25]. The DOCC intervention produced mixed results, with cost savings at 6- and 12-months post-intervention (derived from reduced health care use) but not at 18- and 24-months post-intervention [25,27].

#### Multidisciplinary Primary Care Teams

All three MDT interventions (CYPHP, PC-COCO, SCZ) reported cost-effectiveness analyses (CEA) with CYPHP also including a cost-utility analysis. The CYPHP was not considered cost-effective from a health care perspective with an ICER of £45,586 GBP per QALY at 12 months (compared to WTP of £20,000–30,000 GBP per QALY gained). However, it was reported as cost-effective from a societal perspective, which accounted for caregiver time and work absences (ICER of £22,966 GBP per QALY at 12 months) [24]. The PC-COCO was reported to be marginally cost-effective compared with the control group with an ICER of £175 GBP per 0.1 BMI standard deviation score reduction [22]. The SCZ was reported as cost-effective, with a dominant ICER (reduced hospital use and cost) and total net saving based on modelling, but results were not statistically significant [23].

#### Co-consultation

The LT and SC4CP interventions completed a cost analysis [21,29]. The LT intervention was more expensive than usual care to deliver, costing an additional $37 GBP per clinic session compared with usual training [21]. The SC4CP model was also more expensive than usual care to deliver with model estimates to cost $172 AUD more per child seen in co-consults, compared with usual care [29].

### Health service and clinical outcomes

Primary health service or clinical outcome for each study is shown in Table 2. Five studies reported a statistically significant primary outcome. The LT study showed a statistically significant improvement in guideline adherence to four conditions (constipation, asthma, feverish illness, eczema) during and after the intervention [15]. The PC-COCO study reported a statistically significant reduction in BMI standard deviation score (SDS) compared with the control group [22]. The CAI reported a statistically significant reduction in hospitalisation and ED presentations based upon parent-completed interviews [25]. The ROAD intervention reported a statistically significant reduction in depression symptoms compared with the control group [26] and the DOCC study had a significant improvement to child mental health symptoms and parental stress in the intervention compared with the control group [27].

The ICCC showed no statistically significant difference in child QoL between groups [30]. The SC4CP showed a non-significant reduction in ED presentations and referrals to hospital clinics [29] and the SCZ similarly found no statistically significant difference in ED presentations [23]. Lastly, the CYPHP reported no differences in ED presentations or child quality of life [33].

### Methodological quality

Table 4 reports the results based on the CHEERS criteria [20]. The cost analysis studies (LT, SC4CP and DOCC) were not subjected to the appraisal as they are purely costing studies which do not strictly fit the CHEERS framework for assessment of quality. The methodological quality of studies varied between 54%–88%, with a median of 79%. All studies identified the study population, setting and location health perspective of the economic analysis, time horizon, measurement of outcomes and valuation of resources and costs. Most studies included the currency, price date, and conversion of costs used in the analysis and characterising uncertainty of results. The items that fewest papers complied with were characterising heterogeneity, inclusion of a health economic analysis plan, and engaging with stakeholders who were affected by the study.

**Table 4:** Assessment of methodological reporting quality of included studies using the CHEERS 2022 checklist.


		STUDY	CARTER	HOLLINGHURST	MELYDA	SOLEY-BORI	WOODS	WRIGHT

**Title & Abstract**	1	Title	1	1	1	1	0	1

	2	Abstract	1	1	1	1	1	1

**Introduction**	3	Background and objectives	1	1	1	1	1	1

**Methods**	4	Health economic analysis plan	0	0	0	0	0	0

	5	Study population	1	1	0	1	1	1

	6	Setting and location	1	1	1	1	1	1

	7	Comparators	1	1	1	1	1	1

	8	Perspective	1	1	1	1	1	1

	9	Time horizon	1	1	1	1	1	1

	10	Discount rate	1	1	0	1	0	0

	11	Selection of outcomes	1	1	1	1	1	1

	12	Measurement of outcomes	1	1	1	1	1	1

	13	Valuation of outcomes	1	1	1	1	1	1

	14	Measurement and valuation of resources and costs	1	1	1	1	1	1

	15	Currency, price date, and conversion	1	1	1	1	0	1

	16	Rationale and description of model	NA	NA	1	NA	NA	NA

	17	Analytics and assumptions	NA	NA	1	NA	NA	NA

	18	Characterising heterogeneity	0	0	0	1	0	0

	19	Characterising distributional effects	0	0	0	1	0	0

	20	Characterising uncertainty	1	1	1	1	0	1

	21	Approach to engagement with patients and others affected by the study	1	0	0	0	1	0

**Results**	22	Study parameters	1	1	1	1	0	1

	23	Incremental costs and outcomes	1	1	1	1	0	1

	24	Characterising uncertainty	1	1	1	1	0	1

	25	Characterising heterogeneity	0	0	0	0	0	0

**Discussion**	26	Study findings, limitations, generalizability, and current knowledge	1	1	1	1	1	1

**Other relevant information**	27	Source of funding	1	1	1	1	1	1

	28	Conflicts of interest	1	1	1	1	0	1

**Percentage (%)**			**85**	**81**	**75**	**88**	**54**	**77**


Each item for the selected publication was scored as fulfilling the criteria (“1”), not at all (“0”) or not applicable (NA). Note: Cullen, Hiscock and Yu studies were not subjected to the CHEERS methodological reporting criteria as they are purely costing studies which do not strictly fit the CHEERS framework for assessment of quality as they do not include an effectiveness measure.

## Discussion

### Summary of findings

This review found that paediatric integrated care interventions, particularly care coordination models, may be clinically and economically effective, although the evidence base remains limited in scope and methodological rigor. Of the nine studies included in the review, five were cost-effective, one showed cost-savings and one cost-effective only from a societal perspective.

Although these results are encouraging, several factors affect the reliability of these results. The six studies that reported cost-effectiveness all had limitations affecting the robustness of the results. These included short follow ups, small sample size, and variable methodological quality. Our review underscores the inherent methodological challenges in analysing complex health interventions and evaluating large-scale changes within healthcare systems. This is seen by the multifaceted nature of integrated care interventions, context sensitivity and difficulty in isolating and measuring individual effect.

Overall, care coordination models showed the strongest evidence in terms of economic impact. Care coordination models were also tested using the most robust study designs (mainly RCTs). A possible reason for the strength of the care coordination evidence is that these interventions were well-defined, and effectiveness was largely based on improvement in symptoms for the specific disease targeted. The studies grouped under paediatric MDTs had mixed economic results. Both co-consultation interventions were more costly compared with usual care. Caveats to these results were that both studies were pre-post, pilot studies, had moderate methodological quality and consequently were not powered to determine health outcomes and were not designed to provide estimates of economic gains. More comprehensive analyses of cost-effectiveness are needed. One of these co-consultation interventions, SC4CP, has progressed to a full trial and cost-effectiveness analysis, and results are pending.

There were mixed results regarding the primary health outcome of the studies, with five of nine studies demonstrating a statistically significant improvement in the health outcome. There was a trend towards favourable primary outcomes in interventions that were focused on a specific measure and/or targeting a single disease. Care coordination models had the strongest results, with less consistent findings seen in the MDT and co-consultation models. This follows the trend regarding the more complex interventions reporting less favourable results than specific models.

Out of the seven studies that had a favourable economic evaluation, only four had a positive primary health outcome, illustrating the trade-offs that occur between costs and benefits in a cost-effectiveness ratio. When interpreting results of these studies, both the cost-effectiveness findings and health benefits must be considered together. A further consideration when interpreting economic findings across studies is the variation in willingness-to-pay (WTP) thresholds across jurisdictions. Given that included studies were conducted across three countries (UK, USA, Australia), direct comparison of ICERs should be made cautiously.

The broader, more complex interventions where models of care differed significantly from usual care, such as CYPHP, SC4CP and LT, showed less favourable economic results and change in primary outcomes. These results reflect the current literature regarding the difficulty in analysing complex interventions such as integrated care models and the need for more comprehensive analyses in this space [37]. In these models, it is difficult to capture the full range of potential benefits and savings when the economic evaluation is limited in scope. For example, in the CYPHP model, the economic evaluation only considers four specific health conditions and did not account for the co-consultation aspect of the intervention [24]. Interventions focusing on a single disease or specific measure tend to produce stronger statistical effects because they allow for precise resource allocation and clearer outcome tracking, while more complex interventions introduce additional variables that can dilute the statistical effectiveness [37,38].

Another factor complicating economic evaluations of integrated care is that these programs are not just aimed at improving health outcomes. In line with the quintuple aim framework that drives primary care redesign, integrated care also considers non-health benefits, such as improving patient experience, clinician experience, and health equity [39]. These non-health benefits are much harder to quantify and include in economic evaluations [37]. Many of the programs included in the review were targeting low socioeconomic populations to improve health equity, something that the economic evaluations did not consider. These factors may partially explain the lesser degree of evidence in support of more complex interventions that were targeting systems-level change. Only two of the nine studies used a societal perspective in their economic evaluation, with each producing a positive economic result. Utilising a societal perspective in economic evaluations of integrated care programs should be considered more consistently as it factors in broader productivity benefits that these programs can deliver. Another factor limiting the results is the short-term duration of each study, which does not capture the cost-savings beyond the intervention, potentially making the economic results more conservative.

The methodological quality of the economic evaluations included in the review was moderate and varied between studies. Limitations common to the evaluations were failing to characterise heterogeneity of results, and failure to report the key methodological components required in economic analyses. The moderate quality of economic evaluations limits the ability of these results to be used by policymakers, but it is a common issue previously identified in the reporting of the economic evaluation of integrated care models [40] and other types of health interventions [41,42].

Whilst the substantial heterogeneity between interventions makes it challenging to identify clear trends or draw conclusions, it also reflects the diverse nature of integrated care. Most models were developed in response to local challenges and designed to meet the specific needs of that population. This requires a shift to thinking of integrated care as being a set of practices shaped by the specific context in which it is implemented, rather than a single intervention [43]. The lack of a clear definition of integrated care further complicates the comparison of studies.

### Strengths

This review helps to address the lack of evidence on the economic impact of integrated paediatric primary care models and can help policymakers and service providers consider the economics and sustainability of these interventions. The review also provides greater evidence regarding the clinical effectiveness of paediatric integrated care and brings together a wide variety of interventions and models targeting novel approaches to managing paediatric care. Strengths of the methodology included having two reviewers for screening and full text evaluation and a quality appraisal of the economic evaluation which aided synthesis and interpretation of the complicated economic evaluations.

### Limitations

There were several limitations to the methodology of the review. Only three databases were searched, and only studies in English were included, thus limiting potential studies that could have been included. Although CHEERS was used to assess for reporting quality of the studies, it does not evaluate internal validity. Included studies may have methodological weaknesses not captured by this appraisal. A full search of the grey literature was not conducted, potentially limiting the inclusion of economic evaluations presented in government or other reports. However, hand searching of the references in the included studies and a google search did not reveal any such reports. There was only one reviewer of the quality appraisal of the economic evaluation. Finally, cost data were not adjusted for inflation or converted to a common currency year, limiting direct comparability of economic figures across studies.

## Conclusion

There is encouraging evidence that integrated paediatric care models are cost effective and can improve patient outcomes. However, the utility of comparing integrated care interventions is constrained by the heterogeneity of each intervention and the unclear definition of integrated care. Future studies should focus on increasing the quality of economic evaluations with particular attention to a clear description of the intervention. Inclusion of societal perspectives and non-health benefits of integrated care is also necessary to reflect the holistic nature of the programs and should be an important consideration in future studies. Although this review looked at the economic aspect of integrated care, program managers should always consider the social, family and health benefits they offer. This review also highlights the difficulty in analysing the economic impact of complex health interventions that make significant change to the structure of the healthcare system.

## Additional File

The additional file for this article can be found as follows:

10.5334/ijic.10232.s1 Appendix 1. Full search strategy.

## Data Availability

The datasets used and/or analysed during the current study are available from the corresponding author on reasonable request.
