# Supplementary material for: The Economic Impact of Paediatric Integrated Care in Primary Care: A Rapid Review
Source: Int J Integr Care. 2026 Nov 6;26(4):8. doi: 10.5334/ijic.10232 (PMC13741947; doi:10.5334/ijic.10232)
Supplement: Supplementary file 1 [file ijic-26-4-10232-s1.pdf]

Appendix 1. Full search strategy

Database	Search Number	Search Syntax	Filters	Number of records
**MEDLINE**	#1	Integrate* OR integration OR multidisciplinary OR interprofessional OR “care pathway” OR “care coordination” OR “care transition” OR “collaborative care” OR “cooperative care” OR “coordinated care” OR “shared care” OR “transitional care” OR “continuity of care” OR interdisciplinary OR multiprofessional OR “managed care”	Title/Abstract	924,463
	#2	paediatric* OR pediatric* OR youth* OR teen* OR adolescent* OR "young people" OR child* OR "young adults" OR "child and family"	Title/Abstract	2,279,608
	#3	"General Practice" OR "Family Practice" OR "Primary Health Care" OR "primary health care" OR "general practice" OR "family practice" OR "primary care" OR "general practitioner*" OR "family physician" OR "primary care physician" OR "primary care provider*"	Title/Abstract	257,135
	#4	"Cost-analysis" OR "cost analysis" OR "cost savings" OR "cost-benefit" OR economics OR "cost-efficacy" OR "cost effectiveness" OR "cost consequence" OR "economic evaluation" OR "cost-utility" OR "economic assessment" OR "benefit-cost" OR "health economics" OR "economic impact" OR "cost-effectiveness" OR "program costs"	Title/Abstract	157,120
	#5	1,2,3,4		220
	#6	#5	English<br>1 Jan 2010-Current	162

Database	Search Number	Search Syntax	Filters	Number of records
**CINAHL**	#1	Integrate* OR integration OR multidisciplinary OR interprofessional OR “care pathway” OR “care coordination” OR “care transition” OR “collaborative care” OR “cooperative care” OR “coordinated care” OR “shared care” OR “transitional care” OR “continuity of care” OR interdisciplinary OR multiprofessional OR “managed care”	Title/Abstract	235,379
	#2	paediatric* OR pediatric* OR youth* OR teen* OR adolescent* OR "young people" OR child* OR "young adults" OR "child and family"	Title/Abstract	881,235
	#3	"General Practice" OR "Family Practice" OR "Primary Health Care" OR "primary health care" OR "general practice" OR "family practice" OR "primary care" OR "general practitioner*" OR "family physician" OR "primary care physician" OR "primary care provider*"	Title/Abstract	125.354
	#4	"Cost-analysis" OR "cost analysis" OR "cost savings" OR "cost-benefit" OR economics OR "cost-efficacy" OR "cost effectiveness" OR "cost consequence" OR "economic evaluation" OR "cost-utility" OR "economic assessment" OR "benefit-cost" OR "health economics" OR "economic impact" OR "cost-effectiveness" OR "program costs"	Title/Abstract	123,335
	#5	1,2,3,4		105
	#6	#5	English<br>1 Jan 2010-Current	92

Database	Search	Search Syntax	Filters	Number of

	Number			records
**EMBASE (Ovid)**	#1	Integrate* OR integration OR multidisciplinary OR interprofessional OR “care pathway” OR “care coordination” OR “care transition” OR “collaborative care” OR “cooperative care” OR “coordinated care” OR “shared care” OR “transitional care” OR “continuity of care” OR interdisciplinary OR multiprofessional OR “managed care”	.mp	1,174,495
	#2	paediatric* OR pediatric* OR youth* OR teen* OR adolescent* OR "young people" OR child* OR "young adults" OR "child and family"	.mp	3,212,796
	#3	"General Practice" OR "Family Practice" OR "Primary Health Care" OR "primary health care" OR "general practice" OR "family practice" OR "primary care" OR "general practitioner*" OR "family physician" OR "primary care physician" OR "primary care provider*"	.mp	351,455
	#4	"Cost-analysis" OR "cost analysis" OR "cost savings" OR "cost-benefit" OR economics OR "cost-efficacy" OR "cost effectiveness" OR "cost consequence" OR "economic evaluation" OR "cost-utility" OR "economic assessment" OR "benefit-cost" OR "health economics" OR "economic impact" OR "cost-effectiveness" OR "program costs"	.mp	223,851
	#5	1,2,3,4		148
	#6	#5	English<br>1 Jan 2010-<br>Current	129
